# The juxtamembrane and carboxy-terminal domains of *Arabidopsis* PRK2 are critical for ROP-induced growth in pollen tubes

**DOI:** 10.1093/jxb/ert323

**Published:** 2013-10-17

**Authors:** Xin-Ying Zhao, Qun Wang, Sha Li, Fu-Rong Ge, Liang-Zi Zhou, Sheila McCormick, Yan Zhang

**Affiliations:** ^1^State Key laboratory of Crop Biology, College of Life Sciences, Shandong Agricultural University, Tai’an 271018, China; ^2^Plant Gene Expression Center, USDA/ARS and University of California at Berkeley, Albany, CA 94710, USA

**Keywords:** Actin microfilaments, CRIB, polar growth, receptor kinase, ROP GTPases.

## Abstract

Polarized growth of pollen tubes is a critical step for successful reproduction in angiosperms and is controlled by ROP GTPases. Spatiotemporal activation of ROP (Rho GTPases of plants) necessitates a complex and sophisticated regulatory system, in which guanine nucleotide exchange factors (RopGEFs) are key components. It was previously shown that a leucine-rich repeat receptor-like kinase, *Arabidopsis* pollen receptor kinase 2 (AtPRK2), interacted with RopGEF12 for its membrane recruitment. However, the mechanisms underlying AtPRK2-mediated ROP activation *in vivo* are yet to be defined. It is reported here that over-expression of AtPRK2 induced tube bulging that was accompanied by the ectopic localization of ROP-GTP and the ectopic distribution of actin microfilaments. Tube depolarization was also induced by a potentially kinase-dead mutant, AtPRK2_K366R_, suggesting that the over-expression effect of AtPRK2 did not require its kinase activity. By contrast, deletions of non-catalytic domains in AtPRK2, i.e. the juxtamembrane (JM) and carboxy-terminal (CT) domains, abolished its ability to affect tube polarization. Notably, AtPRK2_K366R_ retained the ability to interact with RopGEF12, whereas AtPRK2 truncations of these non-catalytic domains did not. Lastly, it has been shown that the JM and CT domains of AtPRK2 were not only critical for its interaction with RopGEF12 but also critical for its distribution at the plasma membrane. These results thus provide further insight into pollen receptor kinase-mediated ROP activation during pollen tube growth.

## Introduction

Double fertilization of flowering plants requires targeted delivery of sperm by the pollen tube (Johnson and Preuss, 2002). After landing on compatible stigmatic cells, a pollen grain germinates and extends a tube that penetrates deep into the female tissues to deliver sperm. The cylindrical shape of pollen tubes results from growth that occurs at a restricted surface area along a single axis. This tip growth is made possible through co-ordinated cellular activities, among which the spatiotemporal restriction of active ROP GTPases at the apical plasma membrane is the most critical (Cheung and Wu, 2008; Kost, 2008).

ROPs (Rho GTPases of plants) are homologous to metazoan Rac GTPases. By switching between the GDP-bound ‘off’ state and the GTP-bound ‘on’ state, ROPs regulate diverse developmental and cellular activities through binding to downstream effectors (Yang, 2002; Yalovsky *et al.*, 2008). ROPs regulate the Ca^2+^ gradient, dynamic microfilament (MF) organization, and exocytic trafficking, thus acting as central regulators for cell growth and morphogenesis (Cheung and Wu, 2008; Kost, 2008; Yalovsky *et al.*, 2008). Genetically manipulating the balance between GTP-bound and GDP-bound ROPs compromised the polar growth of pollen tubes (Kost *et al.*, 1999; Li *et al.*, 1999; Fu *et al.*, 2001; Cheung *et al.*, 2003; Gu *et al.*, 2005). A plethora of regulatory proteins are in place to make sure that the ‘ROP’ switch is controlled in a spatiotemporal manner (Yang, 2002; Zhang and McCormick, 2008; Fowler, 2010). Thanks to their sequence conservation during evolution, ROP GTPase activating proteins (RopGAPs) and guanine nucleotide dissociation inhibitors (RhoGDIs) were recognized early on in plants (Molendijk *et al.*, 2001; Klahre *et al.*, 2006; Klahre and Kost, 2006). However, the plant-specific RopGEF (guanine nucleotide exchange factors for ROP GTPases) family was only recently identified (Berken *et al.*, 2005). Except for the PRONE domain for GTP-GDP exchange, RopGEFs contain variable non-catalytic domains at the N- or C-terminus that are suggested to play regulatory roles (Gu *et al.*, 2006; Zhang and McCormick, 2007).

Because receptor-like kinases (RLKs) are major cell sensors for perceiving and relaying diverse extracellular signals into the cytoplasm (De Smet *et al.*, 2009), the discovery that plant RLKs interacted with RopGEFs (Kaothien *et al.*, 2005; Zhang and McCormick, 2007) hinted at an exciting possibility as to how environmental stimuli are interpreted into ROP-dependent intracellular activities (Schiller, 2006). Through interaction with RLKs (Kaothien *et al.*, 2005; Zhang and McCormick, 2007; Duan *et al.*, 2010; Chang *et al.*, 2013), RopGEFs are not only released from auto-inhibition but may also be recruited to the plasma membrane where ROPs are ‘switched on’ to initiate intracellular signalling. Such an RLK-RopGEF interaction was proposed to function as a positive feedback mechanism (Zhang and McCormick, 2008), together with negative feedback from the activities of RhoGDIs and RopGAPs (Hwang *et al.*, 2010), to regulate the dynamic activation of ROPs.

It has previously been shown that a pollen-enriched RLK, AtPRK2a, interacts with the pollen-specific RopGEF12 both *in vitro* and *in vivo* (Zhang and McCormick, 2007). Co-expression of AtPRK2a and RopGEF12 resulted in isotropic tube growth, indicative of ectopic ROP activity (Zhang and McCormick, 2007). Functional loss of *AtPRK2a*, renamed as *AtPRK2* in a recent report (Chang *et al.*, 2013) and adopted here, had insignificant effects on pollen germination. Even when combined with mutations in its putative homologues, pollen germination was only mildly reduced (Chang *et al.*, 2013), suggesting higher order redundancy. Chang *et al.* (2013) further showed that over-expressing *AtPRK2* compromised pollen tube growth and that the kinase domain of AtPRK2 interacted with and phosphorylated RopGEF1 *in vitro*. Together, these results hinted at a critical role of AtPRK2 in ROP-induced growth. However, since *RopGEF1* is depleted in pollen tubes (Pina *et al.*, 2005), the biological relevance of the AtPRK2–RopGEF1 interaction is unclear. In addition, the mechanisms underlying AtPRK2-mediated ROP activation *in vivo*, as well as the cellular consequences for AtPRK2-induced depolarization, are yet to be defined.

Evidence is provided here that the non-catalytic domains of AtPRK2 play a critical role in ROP-induced pollen tube growth through RopGEF12. By comparison with the functional loss of *AtPRK2* and its homologues, whose pollen germination was only mildly reduced (Chang *et al.*, 2013) (see Supplementary Fig. S1 at *JXB* online), over-expression of *AtPRK2* induced depolarized pollen tube growth due to the ectopic distribution of active ROP and of actin microfilaments (MF). Such effects relied on the juxtamembrane (JM) and carboxy-terminal (CT) domains of AtPRK2 but not on its kinase activity. It has also been shown that the JM and CT domains but not kinase activity of AtPRK2 were critical for interacting with RopGEF12 at membranes. In addition, these non-catalytic domains were also essential for the subcellular distribution of AtPRK2. Our results provide evidence that the non-catalytic domains of AtPRK2 are essential for its over-expression effects during pollen tube growth, probably by mediating the AtPRK2-RopGEF12 interaction.

## Materials and methods

### Plant growth and transformation

*Arabidopsis* plants were grown in a 4:1:1 by vol. mix of Fafard 4P:perlite:vermiculite under an 18/6h light/dark cycle at 21 °C. To facilitate phenotypic analysis, the mutant *quartet1-2* (*qrt*) in the Col-0 ecotype was used as the wild type for stable transformation using the floral dipping method (Clough and Bent, 1998). Transgenic plants were selected on MS medium supplemented with 30mg l^–1^ Basta salt (Sigma).

### RNA extraction and RT-PCR

Total RNA from diverse tissues of *Arabidopsis* ecotype Columbia (Col-0) was isolated using the RNeasy Plant miniprep kit according to the manufacturer’s instructions (Qiagen). Reverse transcription was performed using Superscript^TM^ III Reverse Transcriptase with on-column DNase I-treatment (Invitrogen). The primers used in the RT-PCR reactions are as follows: PK1/PK2 for AtPRK2, and PK3/PK4 for AtPRK1. *Arabidopsis ACTIN2* was used as the internal control (Zhang and McCormick, 2007). Primers are listed in Supplementary Table S1 at *JXB* online.

### DNA manipulation

All constructs were generated using Gateway^TM^ technology (Invitrogen) except where noted. Entry vectors for *Arabidopsis* AtPRK2 was generated in pENTRY/SD/D TOPO vector (Invitrogen) backbone by using the primer pair PK5/PK6. The entry vector for CRIB_RIC1_ was generated using the primer pair PK7/PK8. AtPRK2_K366R_ and AtPRK2 deletion mutants (AtPRK2ΔJM, AtPRK2ΔCT, AtPRK2ΔJM-CT) were generated using the Phusion site-directed mutagenesis kit (Finnzyme) according to the manufacturer’s recommendation. The AtPRK2 entry vector was used as templates in mutagenesis. The *Pro*_*LAT52*_-driven fluorescent-fusion expression vectors were generated by LR reactions with LR Clonase III (Invitrogen). Pollen-specific destination vectors were described previously (Zhang and McCormick, 2007). Pollen-specific vectors expressing free YFP or CFP were generated by removing the gateway cassettes from the Ghent vectors (Karimi *et al.*, 2002).

Vectors used in the mating-based Split-Ubiquitin System were generated using *in vivo* recombination as described by Obrdlik *et al.* (2004). The coding sequences of AtPRK2, AtPRK2_K366R_, and AtPRK2ΔJM were amplified using the primer pair PK9/PK10 from the corresponding entry vectors, while AtPRK2ΔCT and AtPRK2ΔJM-CT were amplified using the primer pair PK9/PK11 from the corresponding entry vectors. Primers are listed in Supplementary Table S1 at *JXB* online.

All PCR amplifications were done with Phusion^TM^ hot start high-fidelity DNA polymerase (Finnzyme) with the recommended annealing temperature and extension time and were sequenced using an ABI 3300 sequencer. Sequences were analyzed with Vector NTI (Invitrogen). PCR products were recovered with the QIAquick^®^ PCR purification kit, DNA minipreps were with the QIAprep^®^ Spin miniprep kit, and DNA midipreps were with the Qiagen TIP-100 kit.

### Analysis of pollen development and tube growth

Transient expression assays in tobacco pollen were as described previously (Twell *et al.*, 1989; Kaothien *et al.*, 2005). Images were captured from 2–8h after germination. Each construct or construct combination was tested in three independent bombardments and 100–120 tubes were scored. Transgenic pollen of different developmental stages was obtained by dissecting anthers of different sizes. DAPI and aniline blue staining was according to a previous protocol (Johnson-Brousseau and McCormick, 2004). *Arabidopsis in vitro* pollen tube growth was carried out as described by Boavida and McCormick (2007). All *Arabidopsis* pollen tube growth experiments were repeated at least three times.

Final concentrations of 0.4 μg ml^–1^ BFA (Calbiochem) were added to liquid pollen germination medium after 4h incubation and images were taken 30min after the addition of the inhibitor. Treatment with LatB and oryzalin was performed as described by Zhang *et al.* (2010). LatB was added to the pollen germination medium 2.5h after germination to a final concentration of 1nM. Imaging was done after 1h incubation. Oryzalin was added to germination medium 2.5h after germination to a final concentration of 20 μM. Imaging was done after 1h incubation.

### Microscopy and fluorescence quantification

Imaging was performed using either an Axio Observer microscope (Zeiss, www.zeiss.com) with epifluorescence optics equipped with a CCD camera or using a Leica TCS SP5Ⅱ laser scanning microscope (Leica) with a 488nm argon laser and an LP 500 filter. Images were exported and processed using Adobe Photoshop CS3 (Adobe). Fluorescence intensity for the apical region (areas within 10–15 μm of the apex) of pollen tube was measured with ImageJ. Data were collected from 30–40 transgenic pollen tubes from three independent experiments.

### Protein–protein interaction in yeast

The mating-based Split Ubiquitin System was as described by Obrdlik *et al.* (2004). β-Galactosidase quantification of interactions was done using Chlorophenol red-β-d-galactopyranoside (CNPG) as the substrate according to standard protocols (Clontech). Three biological samples were collected for each bait–prey combination and three technical replicates were performed for each sample. Results shown are means ± standard error (SE).

### Sequence analysis

Protein sequences of AtPRK2 orthologues were retrieved using NCBI protein BLAST (http://blast.ncbi.nlm.nih.gov/Blast.cgi). Functional domains were characterized using the online programs Pfam (http://www.sanger.ac.uk/Software/Pfam/) and SMART (http://smart.embl-heidelberg.de/smart/set_mode.cgi?NORMAL=1). Sequence alignments were done using Vector NTI 10 (http://www.invitrogen.com).

### Accession numbers

*Arabidopsis* Genome Initiative locus identifiers for the genes mentioned in this article are: At2g07040, *AtPRK2*; At5g35390, *AtPRK1*; At1g79860, *RopGEF12*; At2g33460, *AtRIC1*.

## Results

### Over-expression of *AtPRK2* caused depolarized tube growth that was not affected by a kinase-inactivating mutation

**Fig. 1.**
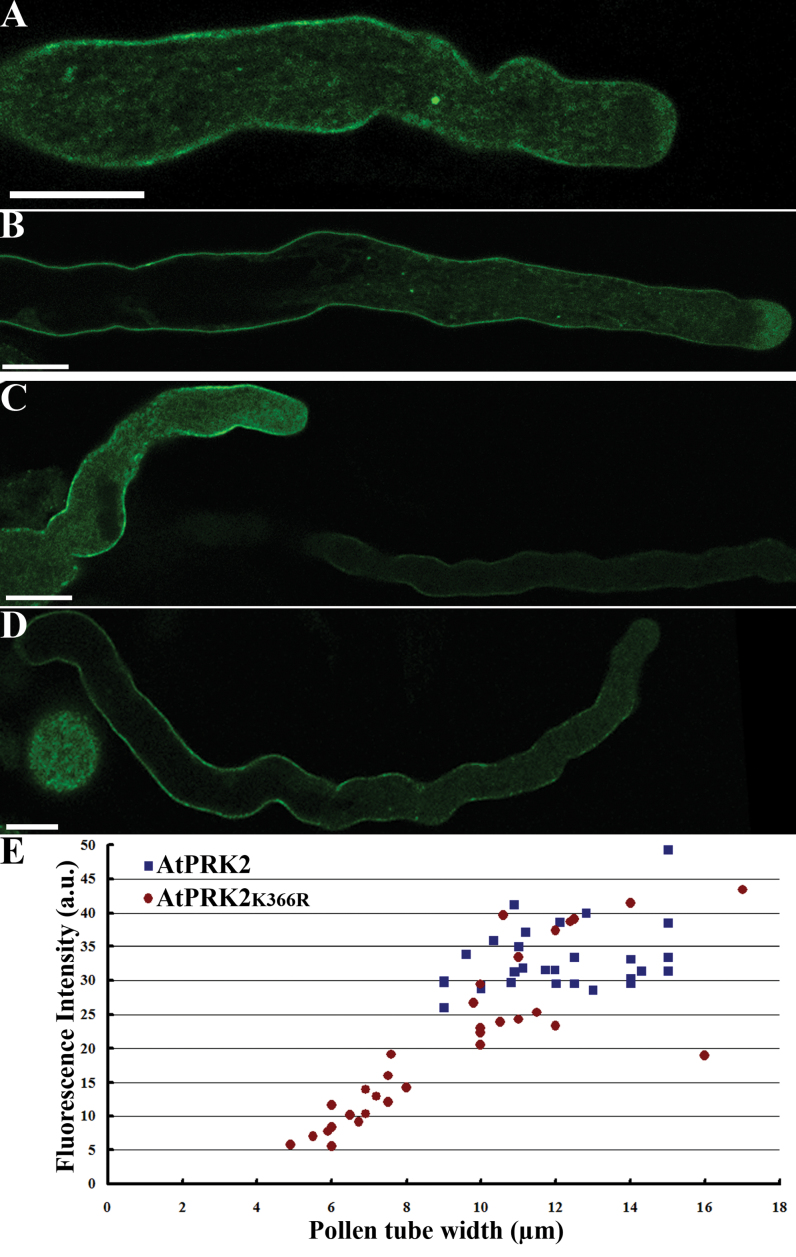
Over-expression of *AtPRK*2 caused depolarized tube growth that was not affected by a kinase-inactivating mutation. *Arabidopsis* pollen tubes over-expressing AtPRK2-GFP (A, B) or AtPRK2_K366R_-GFP (C, D) at an early growth stage (A, C) or late growth stage (B, D). Note that the different levels of transgene expression by the two transgenic pollen tubes in (C) correlate with their tube width. (E) Correlation of expression for transgenic pollen tubes expressing either AtPRK2 or AtPRK2_K366R_. Each data point represents an individual pollen tube for which fluorescence intensity at the apical region (areas within 15 μm to the apex) was measured. a.u., arbitrary fluorescence unit. Data were collected from 31–35 transgenic pollen tubes from three independent experiments. Bar=10 μm.

The mild loss-of-function phenotype of *AtPRK2* and its homologues (Chang *et al.*, 2013) (see Supplementary Fig. S1 at *JXB* online) could be due to redundancy, thus making it difficult to understand gene function by recessive mutations. Therefore, to understand the function of AtPRK2, an over-expression approach, as commonly adopted in pollen biology, was taken (Kost *et al.*, 1998, 1999; Fu *et al.*, 2001; Wu *et al.*, 2001;Cheung *et al.*, 2003; Gu *et al.*, 2005; Hwang *et al.*, 2005, 2010; Zhang and McCormick, 2007; Chang *et al.*, 2013). *Arabidopsis* pollen tubes expressing AtPRK2-GFP driven by the pollen-specific *Pro*_*LAT52*_ (Twell *et al.*, 1990) showed a dosage-sensitive tube-bulging phenotype (Fig. 1A, B, E; see Supplementary Move S1 at *JXB* online), indicating compromised polarity. However, the tube-widening phenotype was most obvious at the early stages of tube growth; transgenic tubes bulged within 100–150 μm of the grain (Fig. 1A) but were fairly normal afterwards (Fig. 1B). This phenotype suggested the presence of an alternative pathway controlling tube polarity during late tube growth.

AtPRK2 was recently confirmed to be an active kinase *in vitro* (Chang *et al.*, 2013). To find out whether the kinase activity of AtPRK2 was critical for its over-expression effect during polarized tube growth, a mutant, AtPRK2_K366R_, was generated that is presumably inactive because an equivalent mutation in a tomato homologue was inactive (Muschietti *et al.*, 1998; Kim *et al.*, 2002). Over-expression of AtPRK2_K366R_-GFP by *Pro*_*LAT52*_ resulted in a dosage-sensitive tube depolarization (Fig. 1C–E) similar to that caused by over-expressing wild-type AtPRK2-GFP (Fig. 1A, B). Therefore it wase concluded that its kinase activity was not essential for AtPRK2-induced tube depolarization.

### Over-expression of *AtPRK2* in *Arabidopsis* resulted in ectopic localization of active ROP and actin microfilaments

**Fig. 2.**
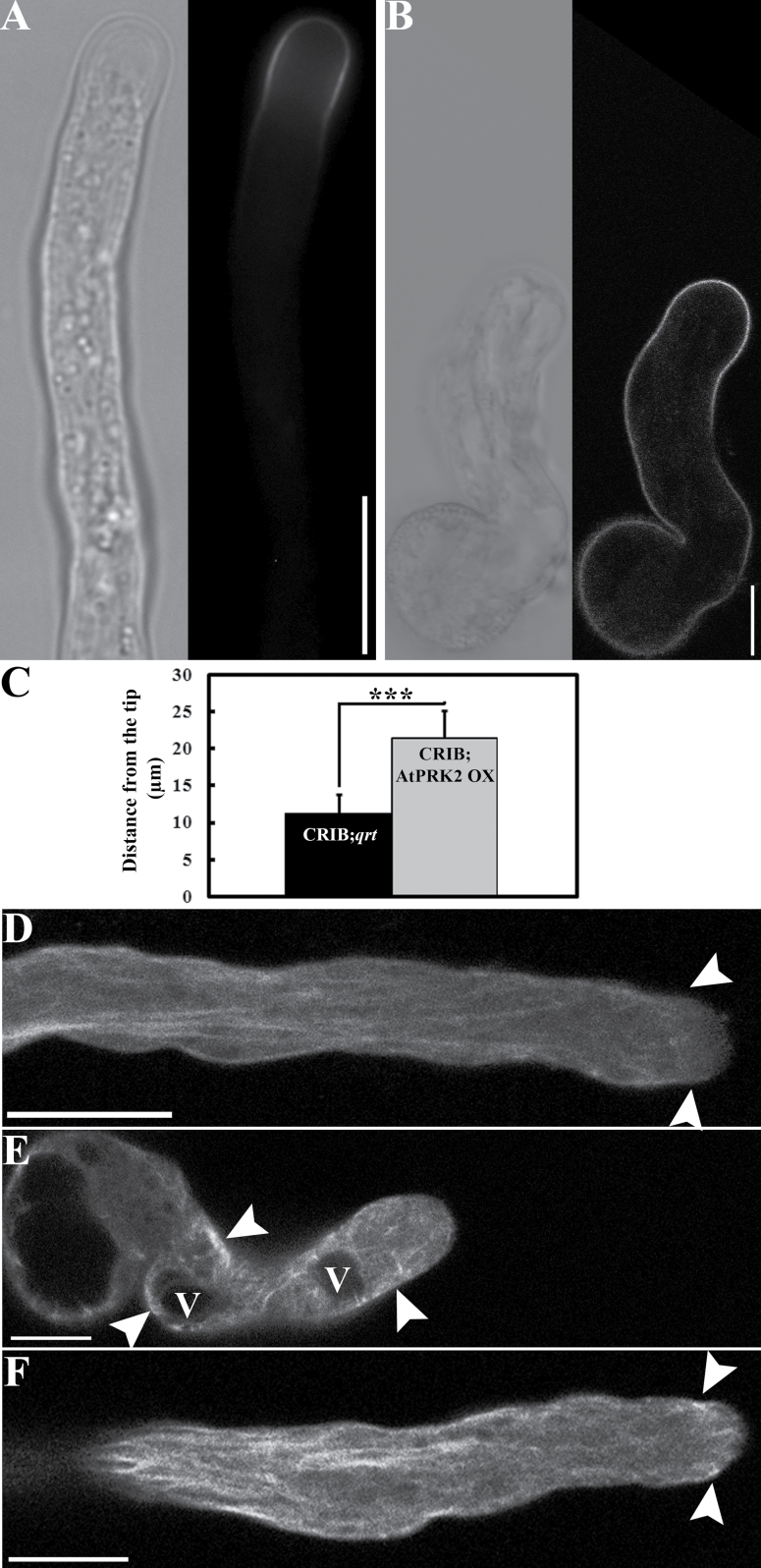
AtPRK2 induced ectopic localization of ROP-GTP and actin MF in pollen tubes. (A-B) *Arabidopsis* pollen tubes expressing CRIB_RIC1_-RFP in *qrt* (A) and in *Pro*_*LAT52*_:AtPRK2-GFP backgrounds (B). Bright field (left) and RFP channel (right) are placed side by side. (C) Quantification of CRIB_RIC1_-RFP distribution in *qrt* tubes (CRIB;*qrt*) or in tubes over-expressing AtPRK2-GFP (CRIB;AtPRK2 OX). Data were collected from three independent experiments, scoring 30–40 pollen tubes in each experiment. Results shown in (C) are given as means ±SE. The distribution of CRIB_RIC1_-RFP is significantly different between *qrt* tubes and tubes over-expressing AtPRK2-GFP as indicated by asterisks (Student’s *t* test, *P* <0.001). (D–F) Pollen tubes expressing mTalin-RFP in *qrt* (D), or in AtPRK2-over-expressing tubes at an early stage (E), and at a later stage (F). V, vacuole. Arrowheads in (D) point to the actin collar just below the apical clear zone, in (E) they point to the short actin cables randomly distributed within the tube, and in (F) they point to the short actin cables penetrating the apical clear zone. Bars=10 μm.

To determine whether depolarization by *AtPRK2* over-expression was due to ectopic ROP activation, a construct expressing an RFP-fused Cdc42/Rac interactive binding (CRIB) domain of RIC1 (CRIB_RIC1_) was generated. RIC1 is a ROP effector (Wu *et al.*, 2001) and it contains a highly conserved CRIB domain which has been used as a biosensor for active ROP (Hwang *et al.*, 2005, 2010). CRIB_RIC1_-RFP expressed in growing pollen tubes of *Arabidopsis* showed only an apical plasma membrane signal (Fig. 2A). By contrast, when CRIB_RIC1_-RFP was co-expressed with AtPRK2, the RFP signal was more extended away from the tip (Fig. 2B, C) than in wild-type tubes, indicating ectopic localization of active ROP.

Because *AtPRK2* over-expression caused the ectopic localization of ROP-GTP, we wondered whether actin MF were also ectopically organized since ROP mediates the dynamic organization of MF in pollen tubes (Fu *et al.*, 2001). To test this idea, mTalin-RFP was introduced into tubes over-expressing *AtPRK2*. mTalin is an MF marker routinely used in pollen tubes to demonstrate MF dynamics (Kost *et al.*, 1998). However, strong expression of mTalin can bundle MF (Ketelaar *et al.*, 2004). To avoid the potential bundling effects by over-expressing mTalin and to make comparisons consistent, wild-type or *AtPRK2* over-expression tubes resulting from crosses were analysed using the same transgenic lines expressing medium level of mTalin-RFP. In such wild-type tubes, MFs were detected as cables in the shank region and as an actin fringe at the base of the apical clear zone (Fig. 2D), as reported previously (Fu *et al.*, 2001). By contrast, over-expressing *AtPRK2* caused the random distribution of short actin cables throughout the pollen tubes at the early stages of growth, that is, in tubes shorter than 150 μm (Fig. 2E). Longitudinal actin cables similar to those in wild-type tubes (Fig. 2D) could be seen in tubes over-expressing *AtPRK2* after prolonged growth (Fig. 2F). However, in those tubes, the actin collar or fringe penetrated to the apex (Fig. 2F) rather than stopping at the base of the apical clear zone, as in the wild type (Fig. 2D).

### Actin MF in pollen over-expressing *AtPRK2* are hypersensitive to LatB and negatively regulate the membrane distribution of AtPRK2 at the apex

**Fig. 3.**
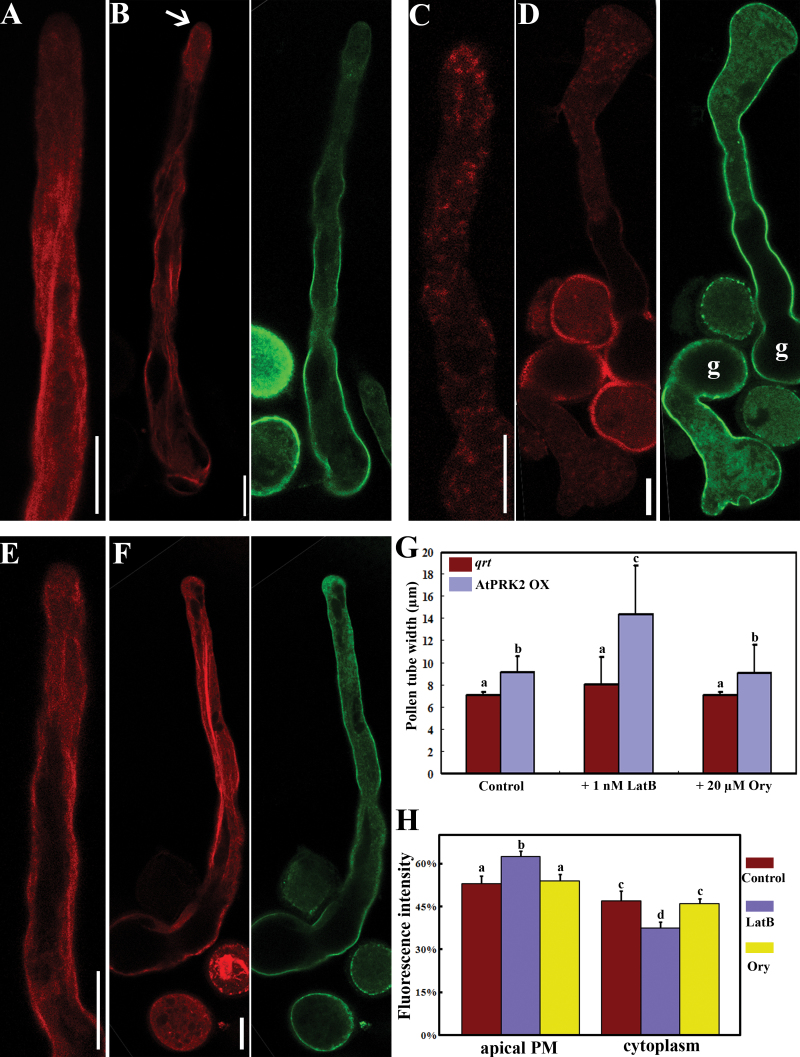
Actin microfilaments (MF) in tubes over-expressing *AtPRK2* are hypersensitive to LatB and negatively regulate the membrane distribution of AtPRK2 at the apex. (A–F) mTalin-RFP transgenic pollen tubes in *qrt* tubes (A, C, E) or in tubes over-expressing *AtPRK2* (B, D, F). These tubes were either treated with DMSO as controls (A, B), or with LatB (C, D), or with oryzalin (E, F). g in (D) indicates pollen grains. RFP channel images (mTalin) and GFP channel images (AtPRK2) are placed side by side for (B), (D), and (F). The arrow in (B) indicates penetrating actin MF at the apex of the tube over-expressing AtPRK2 while the rest of this image shows the distal part of the same tube in the same optical section. Representative images from 80–110 tubes from three independent experiments for each genetic background are shown for (A)–(F). (G) Quantification of pollen tube width treated either with DMSO (control), Latrunculin B (LatB), or oryzalin (Ory). (H) Percentage of fluorescence at the apical plasma membrane (apical PM) or in the apical cytoplasm. The apical region includes areas 10 μm from the apex. Data were collected from 80–110 pollen tubes from three independent experiments for (G) and (H). Samples with different letters (a–d) in (G) and (H) are significantly different from each other by Fisher’s least significant difference (LSD) method. Bars=10 μm for (A) to (F).

That over-expressing *AtPRK2* resulted in disturbance of MF dynamics suggested that tubes over-expressing *AtPRK2* would be hypersensitive to the additional interference of MF polymerization. To test this hypothesis, growing pollen tubes were treated with the MF depolymerization drug Latrunculin B (LatB), while the microtubule depolymerization drug oryzalin was used as a control. The addition of 1nM LatB, a concentration that did not significantly affect the polarity of wild-type tubes (Fig. 3A, C, D, E), resulted in isotropic growth in tubes over-expressing *AtPRK2* (Fig. 3B, D, F). By contrast, oryzalin treatment did not significantly affect tube depolarization caused by *AtPRK2* over-expression (Fig. 3F, G). These results showed that tube depolarization induced by *AtPRK2* is hypersensitive to the pharmacological disruption of actin microfilaments. In addition, it was noted that there was an enhanced accumulation of AtPRK2 at the plasma membrane of the apex when pollen tubes were treated with LatB, compared with its localization in control tubes or oryzalin-treated tubes (Fig. 3H). Because depolymerization of actin MF inhibits endocytosis in pollen tubes (Zhang *et al.*, 2010), this result suggested that dynamic MF polymerization negatively feedback on the membrane distribution of AtPRK2 at the apex, probably through modulating endocytic trafficking.

### Non-catalytic domains of AtPRK2 are critical for its over-expression effects

**Fig. 4.**
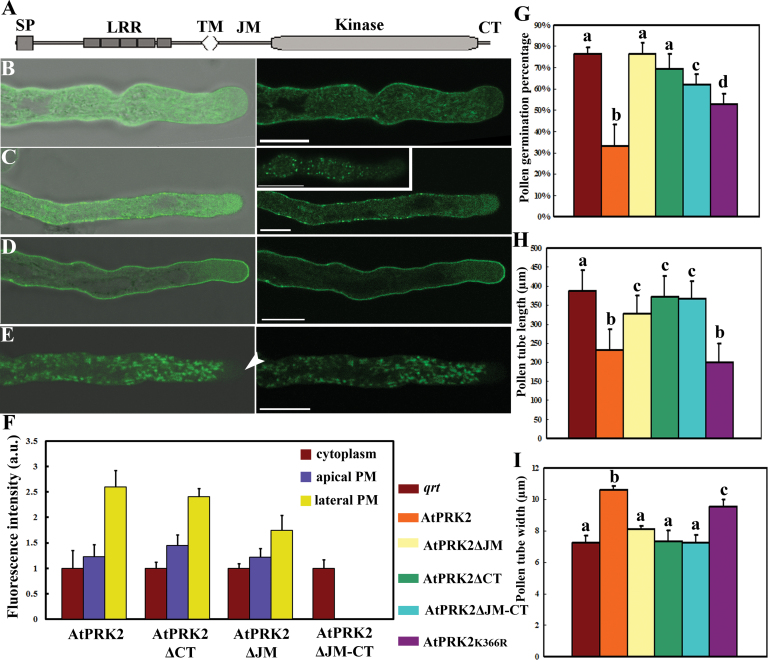
Regulatory domains of AtPRK2 are critical for its subcellular localization and AtPRK2-induced tube depolarization. (A) Schematic illustration of AtPRK2 domain organization. SP, signal peptide; LRR, leucine rich repeats; TM, transmembrane domain; JM, juxtamembrane domain; CT, carboxy-terminal domain. (B–E) *Arabidopsis* pollen tubes over-expressing AtPRK2-GFP (B), AtPRK2*ΔJM-GFP (C), AtPRK2ΔCT-GFP (D), and AtPRK2ΔJM-CT-GFP (E). Because the GFP signals were detected at the plasma membrane in puncta for AtPRK2ΔJM-GFP, an image taken at a different optical plane is shown (C inset) to show the GFP puncta at the plasma membrane. Representative images of 80–-110 tubes from three independent experiments for each genetic background are shown for (B) to (E). Bright field and GFP channel merged images are at the left for (B) to (E). Arrowhead indicates the apex. Bars=10 μm for (B) to (E). (F) Arbitrary fluorescence units (a.u.) at the apical plasma membrane, lateral plasma membrane or in the cytoplasm for AtPRK2 and its mutant variants. The apical plasma membrane was defined as the region 10 μm to the apex while the shank region was defined as the region 30–40 μm away from the apex. Data were collected from 50–60 pollen tubes from three independent experiments. Results are given as means ±SE. (G–I) Germination percentage (G), tube width (H), and tube length (I) of transgenic pollen expressing AtPRK2 and its mutant variants. Germination percentage in (G) was calculated 6h after germination. Tubes over 90 μm long were considered germinated. In total, 1500–3400 pollen grains of each genetic background were analysed. 80–110 tubes from three independent experiments were analysed for tube length in (H) and tube width in (I). Samples with different letters (a–c) in (G), (H), and (I) are significantly different from each other by Fisher’s least significant difference (LSD) method.

A recent study showed that phosphorylation at the JM domain of LePRK2 played a key role in its function during pollen tube growth (Salem *et al.*, 2011). However, AtPRK2 shares no homology at the JM and CT domains with its presumable homologue LePRK2 (Kim *et al.*, 2002), even though RLKs from a few other plant species were homologous to AtPRK2 at the JM and CT domains (see Supplementary Fig. S2 at *JXB* online). To determine whether and how these non-catalytic domains contributed to the functionality of AtPRK2, AtPRK2 truncations were generated in which the JM (AtPRK2ΔJM), the CT (AtPRK2ΔCT), or both (AtPRK2ΔJM-CT) domains were deleted (see Supplementary Fig. S2 at *JXB* online). *Arabidopsis* pollen tubes over-expressing these truncated proteins were compared with pollen tubes over-expressing full-length AtPRK2 (Fig. 4B–E). Deletion of the JM domain or the CT domain abolished the phenotype of AtPRK2 in germination potential (Fig. 4G), tube growth (Fig. 4H), and tube width (Fig. 4I), suggesting that these non-catalytic domains were essential for the AtPRK2-induced ROP activation. Specifically, deletion of the JM domain mis-localized AtPRK2 to patches on the plasma membrane (Fig. 4C, inset) which can also be seen as puncta along the tube plasma membrane (Fig. 4C), rather than the relatively uniform plasma membrane localization of AtPRK2 (Fig. 4B). The CT-deleted AtPRK2 was distributed uniformly at the plasma membrane (Fig. 4D) but did not induce tube depolarization (Fig. 4I). Deletion of both the JM and the CT domains resulted in mis-localization of the protein to motile vesicles excluded from the apical clear zone, suggestive of cytosolic organelles (Fig. 4E; see Supplementary Movie S2 at *JXB* online). It is worth noting that the CT-deleted AtPRK2 was more concentrated at the apical plasma membrane than was wild-type AtPRK2 (Fig. 4F), suggesting altered membrane distribution. These results suggest that the non-catalytic domains were essential for the subcellular localization of AtPRK2 and its activity in inducing tube depolarization.

### AtPRK2-induced tube depolarization depends on its interaction with RopGEF12

**Fig. 5.**
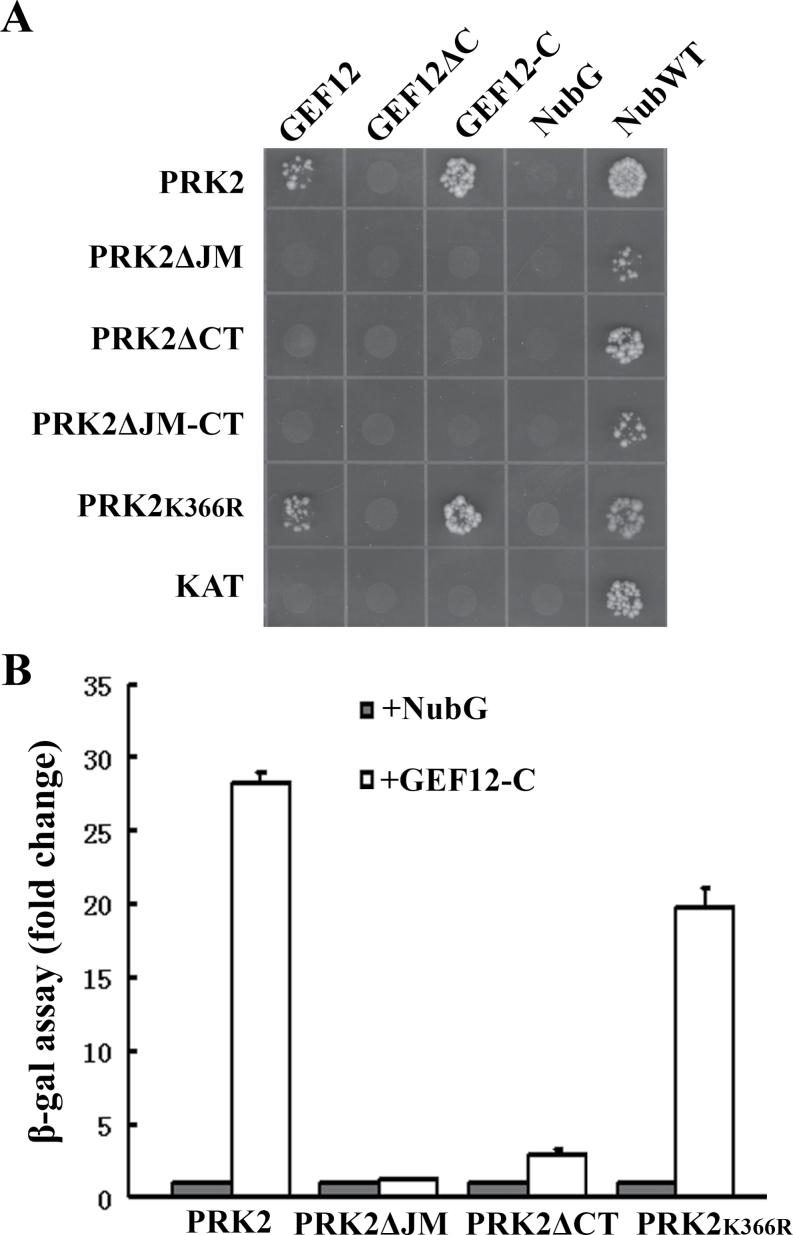
Non-catalytic domains of AtPRK2 are critical for its interaction with RopGEF12 in yeast. (A) Interaction between RopGEF12 and AtPRK2 and its mutant variants on selective medium (–WLUHA). KAT was used as a negative control for the baits. NubWT and NubG were used as positive or negative controls for the prey, respectively. (B) Quantification of protein–protein interactions by β-galactosidase activity. Results are means ±SE of three biological replicates.

It was previously shown that AtPRK2 regulates tube polarity through interacting with RopGEF12 (Zhang and McCormick, 2007). In line with the current findings that the non-catalytic domains of AtPRK2 were essential for AtPRK2-induced tube depolarization, it was hypothesized that these non-catalytic domains might be critical for the interaction between AtPRK2 and RopGEF12 and thus deletions of these domains would render AtPRK2 incapable of inducing ectopic ROP activation. To test this hypothesis, the interaction between the AtPRK2 deletions and RopGEF12 was analysed in the mating-based split ubiquitin system (mbSUS) which detects protein–protein interactions at the plasma membrane. Deletion of the JM domain or the CT domain completely abolished the interaction between AtPRK2 and RopGEF12 (Fig. 5), confirming that the interaction of AtPRK2 with RopGEF12 requires its non-catalytic domains. Unlike deletions of the non-catalytic domains of AtPRK2, the K366R mutation did not abolish its interaction with RopGEF12 although it did show a reduced affinity (Fig. 5), thus excluding the possibility that deletion of the non-catalytic domains rendered AtPRK2 inactive, and by doing so, abolished its interaction with RopGEF12.

**Fig. 6.**
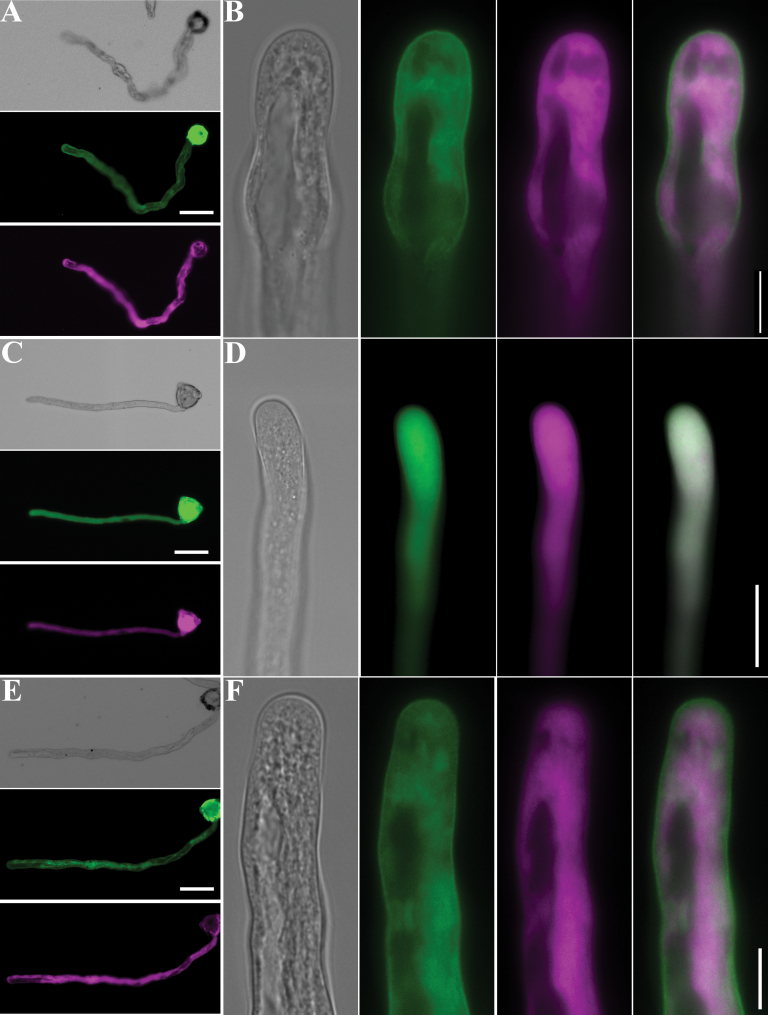
Co-expressing the C-terminal domain of RopGEF12 significantly suppressed polarity defects induced by *AtPRK2* over-expression. (A–F) Tobacco pollen tubes co-expressing AtPRK2-CFP (green) and YFP (magenta) (A, B), YFP-GEF12-C (green) and CFP (magenta) (C, D), or AtPRK2-CFP (green) and YFP-GEF12-C (magenta) (E, F) are shown. Bars=50 μm for (A), (C), and (E), 10 μm for (B), (D), and (F). Representative images from 31–35 transgenic pollen tubes from three independent experiments are shown.

The C-termini of some RopGEFs auto-inhibit GEF activity (Gu *et al.*, 2006; Zhang and McCormick, 2007). It was proposed previously that the interaction between AtPRK2 and the C-terminus of RopGEF12 releases the auto-inhibition and allowing ROP activation (Zhang and McCormick, 2007). If the AtPRK2-RopGEF12 interaction is indeed critical for AtPRK2-induced ROP activation, then an excess of the C-terminal fragment might reduce the depolarized growth caused by AtPRK2 over-expression, due to competitive binding to AtPRK2. To test this hypothesis, a *Pro*_*LAT52*_:YFP-RopGEF12-C construct (RopGEF12_444-515_, designated as GEF12-C) was generated and co-expressed with *Pro*_*LAT52*_:AtPRK2-CFP. Co-expression of AtPRK2-CFP and YFP, as well as co-expression of YFP-GEF12-C and CFP, served as controls. Co-expression of AtPRK2-CFP and YFP showed disturbed pollen tube polarity (Fig. 6A, B). About 30% of the transformed tubes exhibited bulged tips at the apical region (Fig. 6A, B; see Supplementary Fig. S3 at *JXB* online), and another 30% showed signs of changing or changed growth trajectories (Fig. 6A, B; see Supplementary Fig. S3 at *JXB* online). The remaining tubes were wider than tubes transformed with GFP alone (see Supplementary Fig. S3 at *JXB* online). Co-expression of YFP-GEF12-C and CFP did not change tube morphology discernibly (Fig. 6C,D), although tube width and growth was slightly reduced (see Supplementary Fig. S3 at *JXB* online; data not shown). GEF12-C localized in the cytoplasm, as did CFP (Fig. 6C, D). However, co-expressed GEF12-C significantly suppressed both the tube widening and axis change phenotype induced by AtPRK2 over-expression (Fig. 6E, F; see Supplementary Fig. S3 at *JXB* online), suggesting that exogenous GEF12-C competitively inhibited the ectopic ROP activation induced by AtPRK2 over-expression.

## Discussion

A large number of RLKs are encoded in plant genomes (Shiu and Bleecker, 2001*a*, b). Their functions cover a wide spectrum of processes, including cell differentiation and organ development, hormone signalling, plant–microbe interactions, and gametophyte development and interactions (De Smet *et al.*, 2009). The diverse extracellular domains of RLKs ensure specificity in sensing various input signals (Shiu and Bleecker, 2001b) but how these diverse input signals are translated through RLKs into cellular activities is still largely unanswered. Therefore, the discovery that RLKs may regulate ROP activity directly or indirectly through RopGEFs (Kaothien *et al.*, 2005; Zhang and McCormick, 2007; Duan *et al.*, 2010; Humphries *et al.*, 2011; Chang *et al.*, 2013) provides an exciting venue to address how signal interpretation through RLKs acts.

Over-expression of *AtPRK2* compromised pollen tube polarity (Zhang and McCormick, 2007; Chang *et al.*, 2013). Although AtPRK2 was confirmed to be an active kinase by *in vitro* assays (Chang *et al.*, 2013), its over-expression effect does not seem to depend on phosphorylation such that a presumably kinase-dead AtPRK2 (Chang *et al.*, 2013) still induced pollen tube depolarization when over-expressed (Fig. 1). Not surprisingly, bulged pollen tubes caused by *AtPRK2* over-expression contained ectopic ROP-GTP at the plasma membrane (Fig. 2). As a result of ectopic ROP activity, actin microfilaments were ectopically distributed (Fig. 2).

AtPRK2 regulates ROP activation through RopGEFs, either by recruiting RopGEF12 to the plasma membrane (Zhang and McCormick, 2007) or by activation through the phosphorylation of RopGEF1 (Chang *et al.*, 2013). Both mechanisms are used in animal receptor tyrosine kinases (RTKs)-mediated RhoGEF activation (Schiller, 2006). Although the plant-specific RopGEFs are not homologous to their animal counterparts (Berken *et al.*, 2005; Garcia-Mata and Burridge, 2007), the domain organization of RopGEFs suggested similar regulatory mechanisms. The PRONE domain of RopGEFs is responsible for guanine nucleotide exchange (Berken *et al.*, 2005; Gu *et al.*, 2006) while their C-terminal domains, despite being divergent among RopGEF family members, conferred autoinhibition *in vitro* (Gu *et al.*, 2006) and *in vivo* (Zhang and McCormick, 2007; Chen *et al.*, 2011). It was previously shown that AtPRK2 interacts with RopGEF12 through its C-terminal domain (GEF12-C) and by doing so, releases its autoinhibition *in vivo* (Zhang and McCormick, 2007). It is shown here that over-expression of GEF12-C significantly reduced the tube-bulging phenotype caused by *AtPRK2* over-expression (Fig. 6), suggesting ectopic GEF activity induced by AtPRK2 through its interaction with GEF12-C. However, AtPRK2 is relatively uniform along the plasma membrane, even less so in the very apex (Fig. 1), whereas active ROPs, as reflected by the localization pattern of RIC1, are at the apical flank (Fig. 2). An intriguing question to the AtPRK2-RopGEF-ROP hypothesis is how the uniform AtPRK2 can be translated into the restricted ROP-GTP localization. A likely scenario is that different lipid and protein compositions along the plasma membrane of pollen tubes play important roles in AtPRK2 action. Positive effects at the apical flank or negative effects at the shank region would be sufficient for the transition from uniform to restricted localization.

More and more evidence indicates that the JM and CT domains play important roles in regulating the intracellular signalling of plant RLKs. For example, the JM and CT domains of BRI1, the receptor for the plant hormone brassinosteroid, were critical for kinase activation by an autoinhibitory mechanism (Wang *et al.*, 2005; Oh *et al.*, 2012). A phosphorylation site in the rice RLK XA21 was not only important for its autoactivation but also affected its interaction with several cytosolic interactors (Chen *et al.*, 2010). It was also shown that phosphorylation at the JM domain regulated FLS2 internalization (Robatzek *et al.*, 2006). This is consistent with results of a phosphoproteomic study that found that most phosphopeptides within plant RLKs came from either the JM or the CT domains and are generally unique for a single RLK (Nuhse *et al.*, 2004), suggesting that these domains play critical roles in regulating receptor signalling intracellularly. The JM and CT domains of AtPRK2 and its orthologues share little conservation (see Supplementary Fig. S2 at *JXB* online), unlike their kinase domains (Kim *et al.*, 2002). Only a few conserved residues within the JM domain could be identified by aligning AtPRK2 with its orthologues from different plant species (see Supplementary Fig. S2 at *JXB* online). Indeed, the two ser/thr-enriched stretches within the JM domain of LePRK2, acting antagonistically in LePRK2-induced tube growth (Salem *et al.*, 2011), are not present in AtPRK2 (see Supplementary Fig. S2 at *JXB* online Deletion of either the JM or CT domain abolished the interaction of AtPRK2 with RopGEF12 (Fig. 5), as well as its over-expressing effects (Fig. 4), suggesting that the JM and CT domains play essential roles in AtPRK2 functionality, probably through RopGEF12 interaction.

AtPRK2 might initiate ROP activation through different RopGEFs either through interaction or through phosphorylation. RopGEF12 is one of the few pollen-specific or enriched RopGEFs (Gu *et al.*, 2006; Zhang and McCormick, 2007), sharing highly conserved phosphorylation residues within the C-terminal domains whose mutations were shown to release auto-inhibition *in vivo* (Zhang and McCormick, 2007). It was previously shown that over-expression of full-length RopGEF12 did not cause tube depolarization while over-expression of the C-terminal deleted version compromised tube polarity, similar to that caused by over-expression of a constitutively active but membrane association-defective ROP (Kost *et al.*, 1999), although to a lesser extent (Zhang and McCormick, 2007) than that caused by constitutive active ROP1 (Li *et al.*, 1999). By contrast, over-expression of full-length RopGEF1 or any of its truncation mutants containing the complete PRONE domain caused isotropic growth of pollen tubes (Gu *et al.*, 2006), resembling that caused by over-expressing CA-ROP (Kost *et al.*, 1999; Li *et al.*, 1999). These results suggest that distinct regulatory mechanisms exist in the AtPRK2-RopGEF signalling pathway to ensure dynamic ROP activation during the polarized growth of pollen tubes.

## Supplementary data

Supplementary data can be found at *JXB* online.

Supplementary Fig. S1. Functional loss of *AtPRK2* and its close homologue *AtPRK1* reduced pollen germination but did not affect the polar growth of pollen tubes significantly.

Supplementary Fig. S2. Sequence alignment of the non-catalytic domains of AtPRK2 and its related RLKs.

Supplementary Fig. S3. Polarity defects of pollen tubes induced by AtPRK2 are significantly suppressed by co-expressed RopGEF12-C.

Supplementary Movie S1. An *Arabidopsis* pollen tube over-expressing AtPRK2-GFP.

Supplementary Movie S2. An *Arabidopsis* pollen tube over-expressing AtPRK2ΔJM-CT.

Supplementary Table S1. Primers used for RT-PCR.

Supplementary Data

## Abbreviations:

PRK2: pollen receptor kinase 2
ROP: Rho GTPases of plants
CRIB: Cdc42/Rac interactive binding
RopGEF: guanine nucleotide exchange factors for ROP GTPases
JM: juxtamembrane domain
CT: carboxy-terminal domain
RLK: receptor like kinase
MF: microfilaments.
