# Supplementary material for: The juxtamembrane and carboxy-terminal domains of Arabidopsis PRK2 are critical for ROP-induced growth in pollen tubes
Source: J Exp Bot. 2013 Oct 17;64(18):5599–610. doi: 10.1093/jxb/ert323 (PMC3871813; doi:10.1093/jxb/ert323)
Supplement: Supplementary Data [file supp_64_18_5599__index.html]

The juxtamembrane and carboxy-terminal domains of Arabidopsis PRK2 are critical for ROP-induced growth in pollen tubes — The juxtamembrane and carboxy-terminal domains of Arabidopsis PRK2 are critical for ROP-induced growth in pollen tubes — Supplementary Data 

# The juxtamembrane and carboxy-terminal domains of *Arabidopsis* PRK2 are critical for ROP-induced growth in pollen tubes

## Supplementary Data

Data files

**Files in this Data Supplement:**

- Supplementary Data - Supplementary Data
- Supplementary Data - Supplementary Data
- Supplementary Data - Supplementary Data
