# Supplementary material for: The juxtamembrane and carboxy-terminal domains of Arabidopsis PRK2 are critical for ROP-induced growth in pollen tubes
Source: J Exp Bot. 2013 Oct 17;64(18):5599–610. doi: 10.1093/jxb/ert323 (PMC3871813; doi:10.1093/jxb/ert323)
Supplement: Supplementary Data [file supp_ert323_jexbot105874_file001.pdf]

Supplemental Data

The following materials are available in the online version of this article.

Supplemental Figure S1. Functional loss of *AtPRK2* and its close homolog *AtPRK1* reduced pollen germination but did not affect the polar growth of pollen tubes significantly.

Supplemental Figure S2. Sequence alignment of the non-catalytic domains of AtPRK2 and its related RLKs.

Supplemental Figure S3. Polarity defects of pollen tubes induced by AtPRK2 are significantly suppressed by co-expressed RopGEF12-C.

Supplemental Movie S1. An Arabidopsis pollen tube overexpressing AtPRK2-GFP.

Supplemental Movie S2. An Arabidopsis pollen tube overexpressing AtPRK2ΔJM-CT.

Supplemental Table S1. Primers used for RT-PCR.

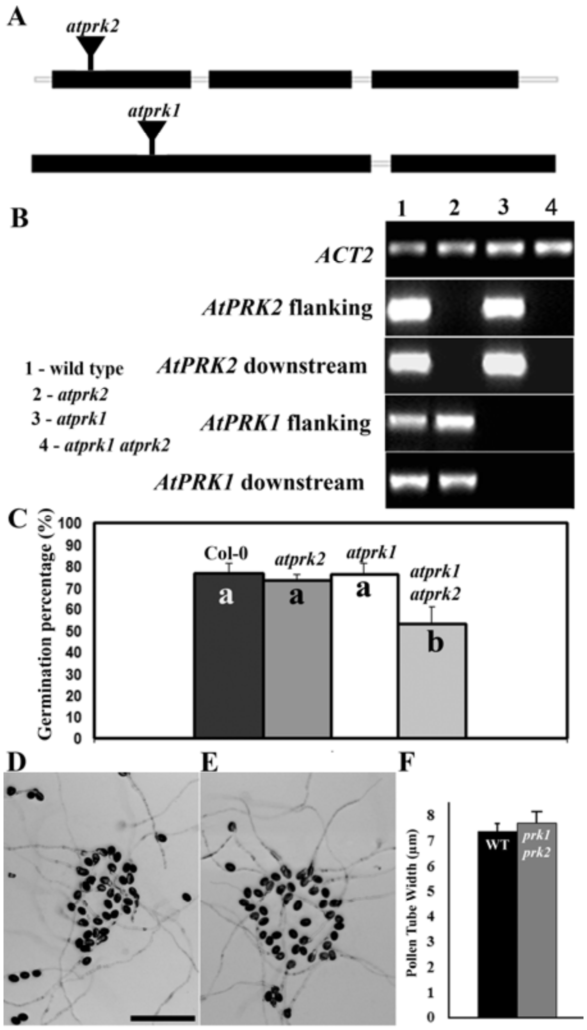

Figure S1. Functional loss of *AtPRK2* and its close homolog *AtPRK1* reduced pollen germination but did not affect the polar growth of pollen tubes significantly.

(A) Schematic representation of *AtPRK2* and *AtPRK1* loss-of-function alleles. (B) Transcript analysis of *AtPRK2* and *AtPRK1* loss-of-function mutants by RT-PCR. *ACT2* was used as the internal control. (C) Quantification of germination percentage. Results shown in (C) are given as means ± standard error (SE). Bars with different letters are significantly different from each other by Fisher's least significant difference (LSD) method. Representative pollen tubes germinated *in vitro* are shown for wild type (D) and for the double mutant *prk1 prk2* (E). The diameter at the subapical region of pollen tubes was measured as the pollen tube width. Data were collected from three independent experiments involving 30-40 pollen tubes in each replicate. Results shown in (F) are given as means ± standard deviation (SD). Bar = 200 μm.

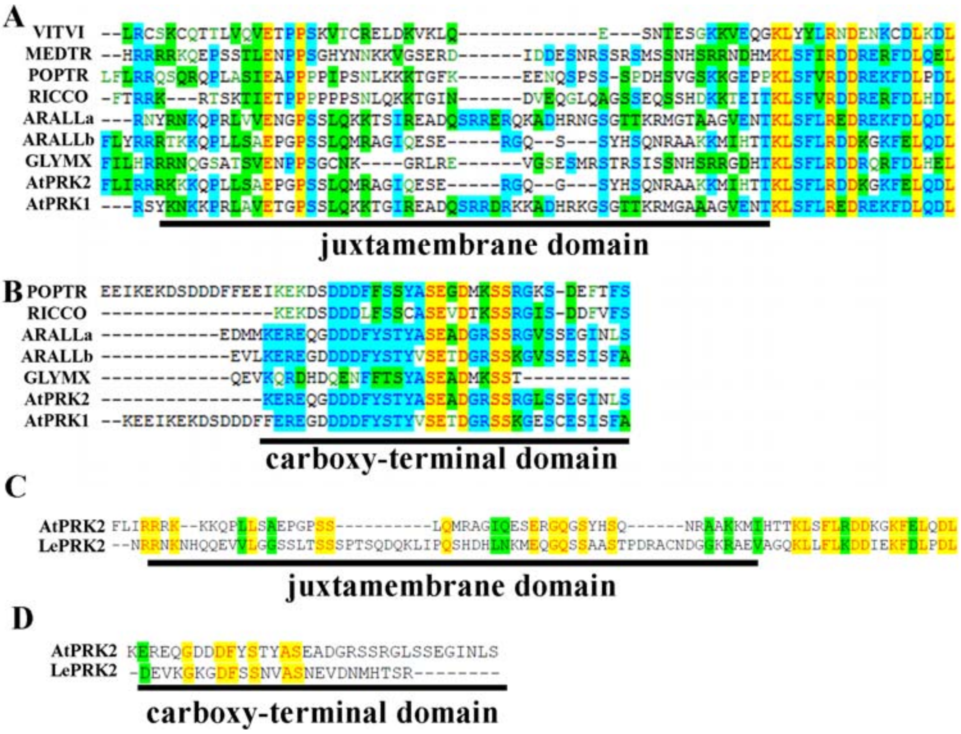

Figure S2. Sequence alignment of the non-catalytic domains of AtPRK2 and its related RLKs.

(A-B) Alignment of the juxtamembrane domains (B) or the carboxy-terminal domains (B) of putative AtPRK2 homologs. Sequences underlined were deleted from AtPRK2 to generate AtPRK2ΔJM or AtPRK2ΔCT, respectively. (C-D) Alignment of the juxtamembrane domains (C) or the carboxy-terminal domains (D) of AtPRK2 and LePRK2. AtPRK2 orthologs used for sequence alignment of the regulatory domains have the following NCBI reference numbers: XP_002883746.1 for ARALLa, XP_002868416.1 for ARALLb, XP_002309026.1 for POPTR, XP_002531377.1 for RICCO, XP_003524427.1 for GLYMA, XP_003588623.1 for MEDTR, and XP_002266187.2 for VITVI.

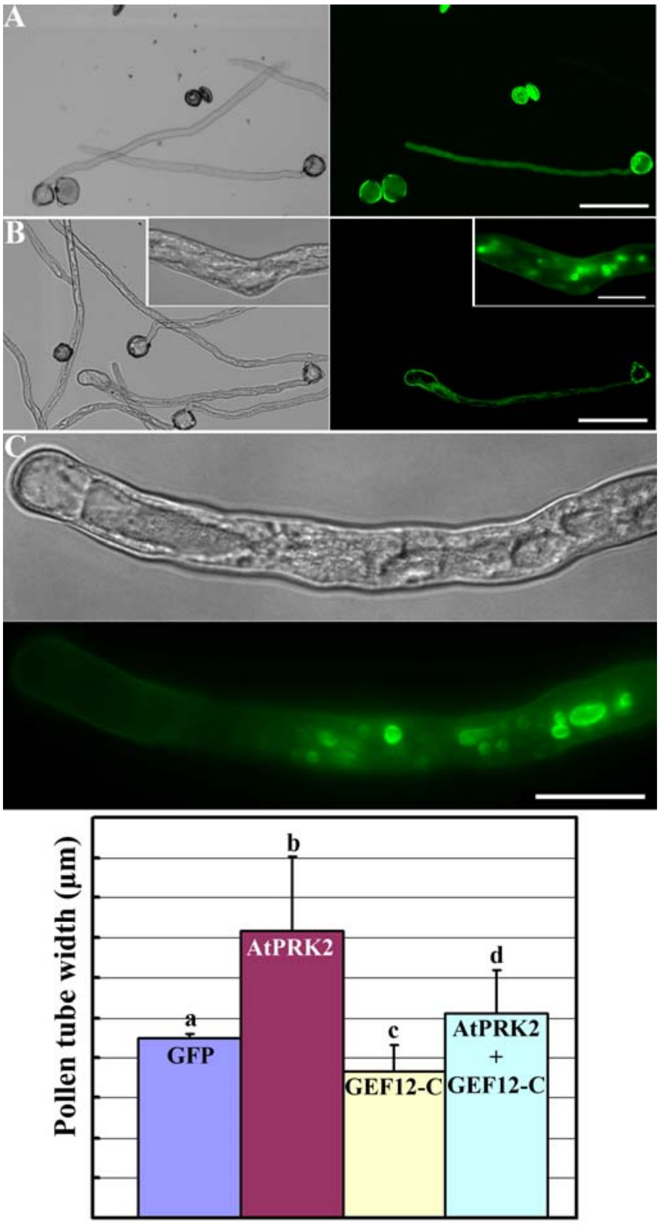

Figure S3. Polarity defects of pollen tubes induced by AtPRK2 are significantly suppressed by co-expressed RopGEF12-C.

(A-B) Pollen tubes transiently expressing free GFP (A), AtPRK2-GFP (B), or AtPRK2-GFP treated with 5 μg/ml BFA for 30 min (C). Inset in (B) shows a representative tube in which AtPRK2 was detected both at the plasma membrane and on cytosolic ring-shaped compartments. Bright field images and GFP channel images are placed side by side. Bars = 100 μm for (A-B), 20 μm for (B) inset and (C). (D) Quantification of tube width by overexpressing free GFP, AtPRK2-YFP, CFP-GEF12-C or co-expressing AtPRK2-YFP and CFP-GEF12-C in tobacco pollen

tubes. Data were collected from 20-30 pollen tubes from at least two independent experiments. Results shown in (D) are given as means ± standard deviation (SD). Samples with different letters (a, b and c) are significantly different from each other by Fisher's least significant difference (LSD) method.

Movie S1. An Arabidopsis pollen tube overexpressing AtPRK2-GFP showing motile punctate vesicles. Bar = 20 μm.

Movie S2. An Arabidopsis pollen tube overexpressing AtPRK2ΔJM-CT. 10 frames of 0.55 μm Z-stack images were collected and outputted at 2 frames/second. Bar = 10 μm.

Table SI. Primers used for RT-PCR.

**Oligos**	**5'-3' sequences**
PK1	AATGGCGTATCAGAGACCGAAA
PK2	GGACTTTTGACCAAGGACGTTG
PK3	AGGCATTGTCGGGTTTGACA
PK4	CTGTTTCCAGCGGTTTTCCA
PK5	CACCATGGAATCCAAATGTCTCATGTTCG
PK6	TCATGACAAGTTAATTCCCTCACTTGATAGT
PK7	CACCATGGCGACGACAATG
PK8	GATAATATCGTTACAGG
PK9	ACAAGTTTGTACAAAAAAGCAGGCTCTCCAACCACCAATGGAATCCAAATGTCTGTCTCATG
PK10	TCCGCCACCACCAACCACTTTGTACAAGAAAGCTGGGTAtGACAAGTTAATTCCCTCACTTG
PK11	TCCGCCACCACCAACCACTTTGTACAAGAAAGCTGGGTACATCTTCTCAACAGCCTCTC
